# Expression of Fucosyltransferase 4 (*FUT4*) mRNA Is Increased in Endometrium from Women with Endometriosis

**DOI:** 10.3390/jcm11195606

**Published:** 2022-09-23

**Authors:** Marta Żeberkiewicz, Anna Hyc, Anna Iwan, Aneta Zwierzchowska, Aneta Ścieżyńska, Ilona Kalaszczyńska, Ewa Barcz, Jacek Malejczyk

**Affiliations:** 1 Department of Histology and Embryology, Medical University of Warsaw, Chałubińskiego 5, 02-004 Warsaw, Poland; 2 Postgraduate School of Molecular Medicine, Medical University of Warsaw, 02-091 Warsaw, Poland; 3 Chair and Clinic of Gynecology and Obstetrics, Faculty of Medicine, Collegium Medicum, Cardinale Stefan Wyszyński University, 04-749 Warsaw, Poland; 4 Department of Obstetrics and Gynecology, Multidisciplinary Hospital Warsaw-Miedzylesie, 04-749 Warsaw, Poland; 5 Diagendo Ltd., 05-504 Bobrowiec, Poland

**Keywords:** endometriosis, endometrium, fucosyltransferase 4 (*FUT4*), progenitor cells, disease marker

## Abstract

Endometriosis is a common gynecological disorder defined as the presence of endometrial-like tissue (glands and stroma) outside the uterus. The etiopathogenesis of endometriosis is still poorly recognized. It is speculated that stage-specific embryonic antigen 1 (SSEA-1)-positive stem-like glandular epithelial cells may contribute to the development of the disease. The synthesis of SSEA-1 is mediated by fucosyltransferase 4 encoded by the *FUT4* gene. Therefore, this study aimed to evaluate the specific expression of *FUT4* mRNA in biopsies of the endometrium from women with and without endometriosis. *FUT4* mRNA levels were examined in 49 women with laparoscopically confirmed endometriosis and 28 controls by means of quantitative reverse-transcription polymerase chain reaction (qRT-PCR). The expression of *FUT4* mRNA was significantly increased in the endometrium of patients with endometriosis when compared to the controls (*p* < 0.0001). Expression of *FUT4* mRNA in the endometrium was correlated with the severity of endometriosis (r_s_ = 0.5579, *p* < 0.0001); however, there were no differences in endometrial *FUT4* mRNA expression when comparing endometriotic lesions from various locations. The discriminatory ability of *FUT4* mRNA expression was evaluated by receiver-operating characteristics (ROC), which showed high statistical significance (AUC = 0.90, *p* < 0.0001), thus indicating that an increased level of endometrial *FUT4* mRNA may serve as a specific marker for endometriosis.

## 1. Introduction

Endometriosis is a common estrogen-dependent gynecological disorder affecting approximately 10% of women of reproductive age. The disease is defined as the presence of endometrial glands and stroma outside the uterus, most frequently in the ovaries and peritoneal cavity. Clinical manifestations may include dysmenorrhea, dyspareunia, chronic pelvic pain and infertility. Endometriosis is associated with chronic pelvic inflammation and may account for up to 50% of female infertility [1,2,3,4,5]. The disease may significantly affect one’s quality of life and, therefore, constitutes an important social, socioeconomic and clinical problem.

The etiopathogenesis of endometriosis is still poorly understood [5,6,7,8]. According to the most commonly accepted theory, Sampson’s retrograde menstruation theory, endometriosis results from shed endometrial cells that survive in the peritoneal cavity, undergo implantation and form ectopic foci of endometrial-like tissue [9]. The mechanisms leading to the survival, implantation and growth of the ectopic endometrial cells may include decreased apoptosis, resistance to eradication by the local immune system, increased adhesiveness, invasiveness, proangiogenic potential and epithelial-to-mesenchymal transition [6,10,11,12,13,14]. 

There is a growing bulk of evidence that the stem-like precursor cells may contribute to the development of endometriotic lesions [15,16,17,18,19,20,21]. These may include mesenchymal stem-like cells that have been identified and characterized among endometrial stromal cells [19,20,22,23,24,25]. Of special interest, however, are epithelial precursor cells expressing N-cadherin (NCAD/CDH2) and/or stage-specific embryonic antigen 1 (SSEA-1/CD15) that were recently described in the basal glandular endometrial epithelium, where they seem to be responsible for the regeneration of endometrial glands following menstruation [26,27]. The NCAD^+^ cells are present in the most basal parts of the glands and gradually change their phenotype to the SSEA-1^+^ cells [15]. The SSEA-1^+^ cells display higher activity of telomerase and express nuclear SOX9 and β-catenin, thus suggesting activation of the WNT pathway [27]. SSEA-1 and SOX9 expression were also identified in ectopic endometrial cells. Most recently, it has been reported that the numbers of SSEA-1^+^ and SOX9^+^ glandular epithelial cells are significantly increased in the eutopic endometrium of women with endometriosis, indicating that they may contribute to the formation of ectopic lesions [28].

SSEA-1, better known as Sialyl-Lewis^X^ (SLeX) or CD15, is a tetrasaccharide carbohydrate composed of sialic acid, fucose and N-acetyllactosamine, which is usually attached to the cell surface by O-glycans [29]. SLeX is involved in embryonic development [30,31], and in adult humans, SLeX plays an essential role in oocyte fertilization [32,33], leukocyte interactions and homing, as well as in the induction of inflammation [34]. Additionally, SLeX is involved in cancer progression, metastasis and angiogenesis [35]. The synthesis of SLeX is mediated by fucosyltransferases 4 and 9 (*FUT4*, 9), which transfer fucose to N-acetyllactosamine polysaccharides [36]. The *FUT4* gene, encoding a 41 kDa protein with α(1,3)-fucosyltransferase activity [37], is located on the long arm of chromosome 11 in position 11q21 [38].

Although endometriosis appears to be associated with increased numbers of SSEA-1/SLeX^+^ cells in the eutopic endometrium, it is highly plausible that this phenomenon may be related to the upregulated expression of the *FUT4* gene. Therefore, the present study was aimed at comparing the expression of specific *FUT4* mRNA in the endometrium of control women and women with endometriosis.

## 2. Materials and Methods

### 2.1. Patients and Material Collection

The study group consisted of 77 women who were admitted to the 1st Department of Obstetrics and Gynecology, Medical University of Warsaw, Poland between January 2010 and December 2015. The endometriosis group consisted of 49 patients in whom the disease was confirmed by laparoscopic and histological examinations. The severity of endometriosis was classified according to the revised American Fertility Society (rAFS) criteria [39]. The control group consisted of 28 women without any clinical or sonographic symptoms or signs of endometriosis who were subjected to the removal of a cervical polyp or underwent cervical biopsy due to an abnormal Pap smear result. Women in whom histopathological examination revealed cervical dysplasia grade 2+ or dysplasia within the cervical polyp were not included. All patients and control subjects had normal menstrual cycles, none of them suffered from any other chronic endocrine or autoimmune disorder and they had not been subjected to hormonal pharmacological treatment for at least 3 months prior to the study. The phase of the menstrual cycle was established based on the date of the last menstrual bleed and the mean cycle duration, as well as being confirmed by pelvic ultrasound examination. Written informed consent was obtained from all participants according to the guidelines of the Declaration of Helsinki and the study was approved by the Institutional Bioethical Review Board of the Medical University of Warsaw, Poland (ref. no. KB/223/2009 and KB/59/2012). The demographic and clinical characteristics of the patients from the endometriosis and control groups are presented in Table 1. As can be seen, there were no significant differences between the endometriosis and control groups, except for the parity and infertility status. 

Tissue samples were collected from the participants during the mid-proliferative and mid-secretory phase of the menstrual cycle directly before surgical intervention. Endometrial tissue samples were obtained by an aspiration biopsy with a Pipelle^®^ catheter (Pipelle de Cornier, Laboratoire C.C.D., Paris, France). Collected specimens were immediately placed in 5 volumes of RNAlater^TM^ solution (TermoFisher Scientific, Waltham, MA, USA), stored at 4 °C for one day and then stored frozen at −70 °C until RNA isolation was performed.

### 2.2. RNA Isolation and Quantitative Reversed-Transcription Polymerase Chain Reaction (qRT-PCR) Analysis

The total RNAs from the endometrial tissue samples (5 mm^3^) were isolated and purified using a NucleoSpin^®^ miRNA Kit with rDNase digestion for removal of genomic DNA (Macherey-Nagel GmbH and Co. KG, Düren, Germany) according to the detailed protocol provided by the manufacturer. The quantity and quality of the isolated RNA were evaluated spectrophotometrically using an ND-2000 Spectrophotometer NanoDrop2000 with software for nucleic acid analysis (ThermoFisher Scientific, Waltham, MA, USA).

A total of 2 μg of large RNA was reverse-transcribed using a High Capacity cDNA Reverse Transcription Kit (Applied Biosystems, Cheshire, UK) according to the manufacturer’s protocol. Briefly, 2 μL of 10 × RT buffer, 0.8 μL of 25 × dNTP Mix, 2 μL of 10 × Random Primers, 1 μL of the Multiscribe Reverse Transcriptase, 4.2 μL of nuclease-free water and 10 μL of large RNA sample were used per reaction. The reaction was run in an Eppendorf Mastercycler gradient with the following conditions: 25 °C for 10 min, 37 °C for 120 min and 85 °C for 5 s. The cDNA samples obtained were stored at −20 °C until qRT-PCR assays were performed.

qRT-PCR was performed in an ABI PRISM 7500 system (Applied Biosystems, Waltham, MA, USA) using 96-well optical plates. For mRNA expression analysis, Hs01106466_s1 for *FUT4* and Hs99999905_m1 for *GAPDH*, FAM-labeled TaqMan probes (Applied Biosystems) were used. Reactions were run in a total volume of 20 μL with TaqMan Universal Master Mix, the appropriate specific primer set, MGB probe and 50 ng of cDNA template. All qRT-PCR assays were carried out under universal conditions with an initial denaturation at 95 °C for 10 min, followed by product amplification through 50 successive cycles of 95 °C for 15 sec and 60 °C for 1 min. Each sample was run in triplicate. The *FUT4* mRNA data were expressed relative to the *GAPDH* mRNA. The data analysis was carried out with Sequence Detection System (SDS) v1.2 software (Applied Biosystems) using the ΔCt method, and the relative mRNA expression is shown as 2^−ΔCt^.

### 2.3. Statistical Analysis

All statistical analyses and graphical presentations were generated using GraphPad Prism 8.2.0 (GraphPad Software, San Diego, CA, USA). The normality of the data distribution was checked by tests included in the GraphPad Prism package. Differences between groups were determined by Student’s *t*-test or the Mann–Whitney *U*-test when applicable. The Kruskal–Wallis test followed by Dunn’s multiple comparison test was used when comparing more than two groups. The two-tailed Spearman’s correlation coefficient (r_s_) was used for correlation analyses. Generation of a receiver-operating characteristics (ROC) curve and calculation of the area under the curve (AUC) and 95% confidence intervals (95% CI) were used to assess the predictive power of *FUT4* mRNA evaluation.

The results are shown as the mean ± SD or median with the interquartile range. A *p*-value < 0.05 was considered statistically significant.

## 3. Results

The relative levels of *FUT4* mRNA expression in eutopic endometrium samples from the control group and women with endometriosis were compared and analyzed with regard to the phase of the menstrual cycle, the stage of disease and the lesion localization.

**Figure 1:**
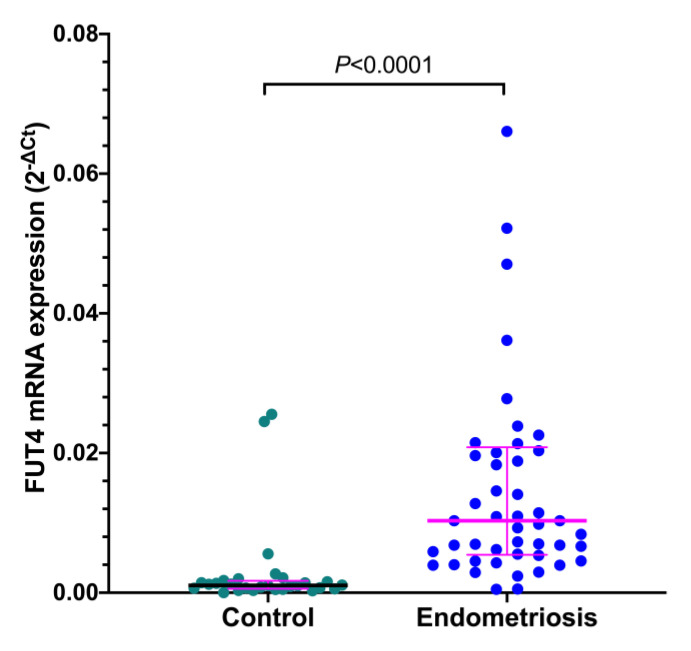
**Expression of *FUT4* mRNA in the endometrium of control women and women with endometriosis.** Expression of *FUT4* mRNA was evaluated by qRT-PCR in the endometrium of control women (*n* = 28) and the endometrium of women with endometriosis (*n* = 49). The data are shown as scatterplots with the median and interquartile range. Samples from the endometriosis group displaying relative expression values over 0.08 (*n* = 3) are not included in the graph. Statistically significant differences between groups were computed by the Mann–Whitney *U*-test.

qRT-PCR analysis revealed that the relative *FUT4* mRNA expression was significantly higher in eutopic endometrium samples from women with endometriosis compared to the control group (*p* < 0.0001) (Figure 1). There were no significant differences in *FUT4* mRNA expression regarding the phase of the menstrual cycle in the endometrium samples from control subjects and patients with endometriosis (data not shown).

**Figure 2:**
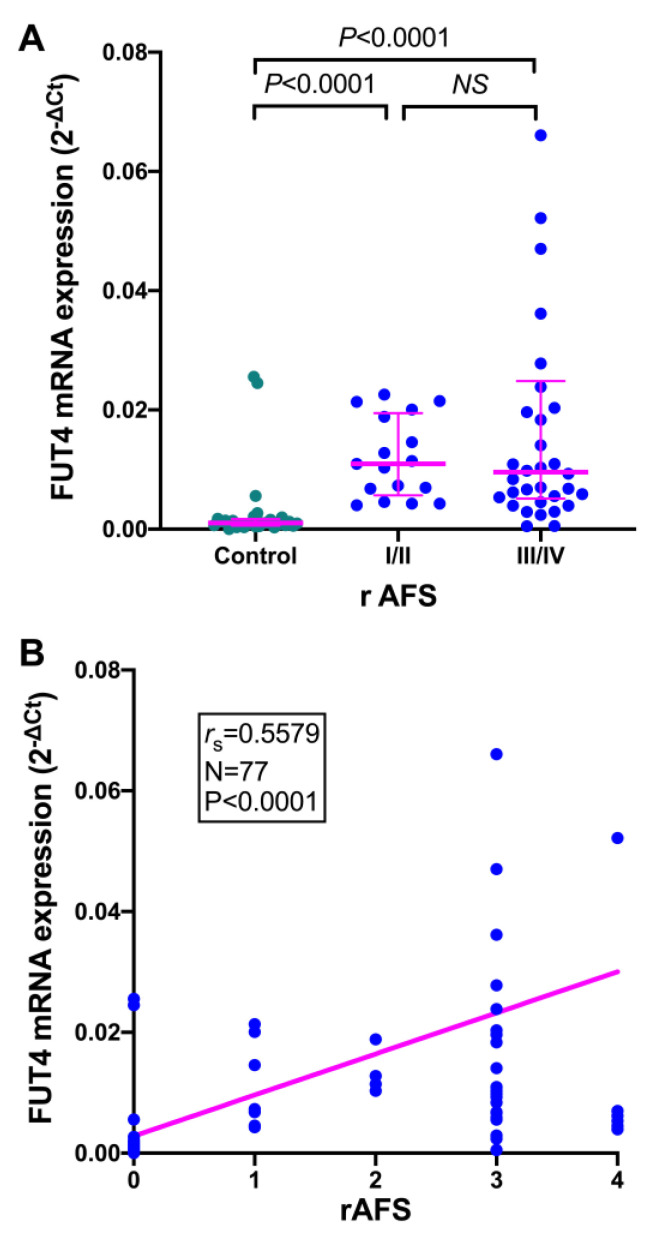
**Expression of *FUT4* mRNA in the endometrium relative to the stage of endometriosis.** (**A**) Expression of *FUT4* mRNA in the endometrium from patients with minimal/mild (I/II) (*n* = 14) and moderate/severe (III/IV) (*n* = 35) stages of endometriosis compared to the endometrium from control women (*n* = 28). Data are shown as scatterplots with the median and interquartile range. Statistical differences between groups were computed by the Kruskal–Wallis test and Dunn’s post hoc test. (**B**) Correlation between *FUT4* mRNA and the rAFS score of the disease. The results are shown as scatterplots with a regression line. Samples from the endometriosis group displaying relative expression values over 0.08 (*n* = 3) are not included in the graph. Spearman’s correlation coefficients (r_s_), their *p*-values and the number of cases (*n*) are shown in an inset.

Analysis of the association between *FUT4* mRNA expression in the endometrium and the stage of endometriosis showed that *FUT4* mRNA expression was significantly upregulated in samples collected from patients with both minimal/mild and moderate/severe stages of disease when compared with the control endometrium samples (Figure 2A). We did not discover any significant differences between patients with less or more advanced stages of endometriosis. Nevertheless, Spearman’s rank correlation analysis revealed that there was a statistically significant positive association (r_s_ = 0.5579, *p* < 0.0001) between *FUT4* mRNA levels and the disease severity (Figure 2B).

**Figure 3:**
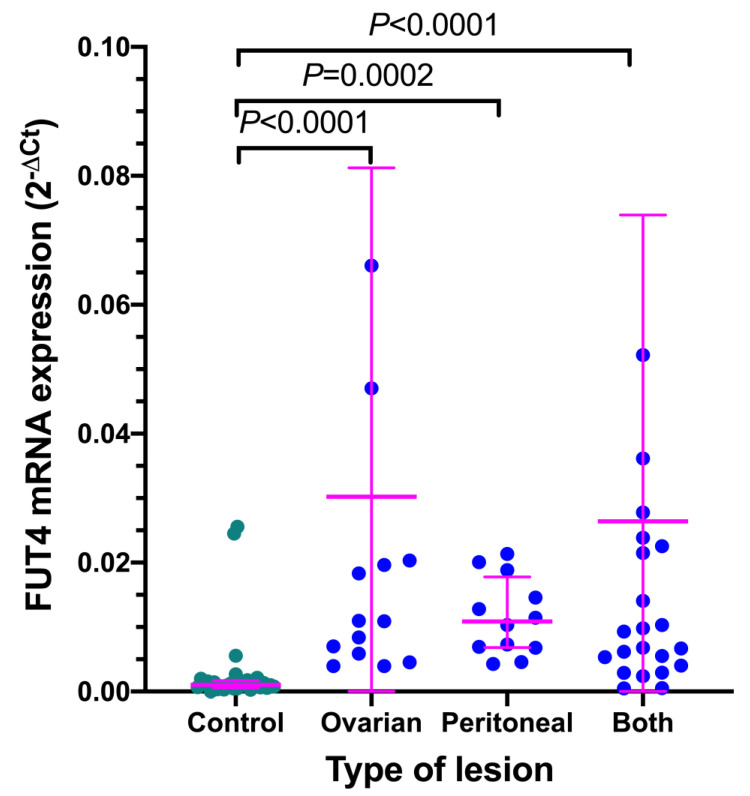
**Expression of *FUT4* mRNA in the eutopic endometrium from women with endometriosis in relation to the localization of ectopic endometriotic lesions.***FUT4* mRNA levels were compared in patients with different lesion locations. The normal endometrium from control women served as a control. All data are shown as scatterplots with median and interquartile ranges. Samples from the endometriosis group displaying relative expression values over 0.08 (*n* = 3) are not included in the graph. Statistical differences between groups were computed by the Kruskal–Wallis test and Dunn’s post hoc test.

Localization of endometriotic lesions (ovarian, peritoneal or both) had no impact on the expression of *FUT4* mRNA in the endometrium of women with endometriosis. Irrespective of localization in all cases, the *FUT4* mRNA levels were significantly higher compared to the endometrium samples from the control group (Figure 3).

**Figure 4:**
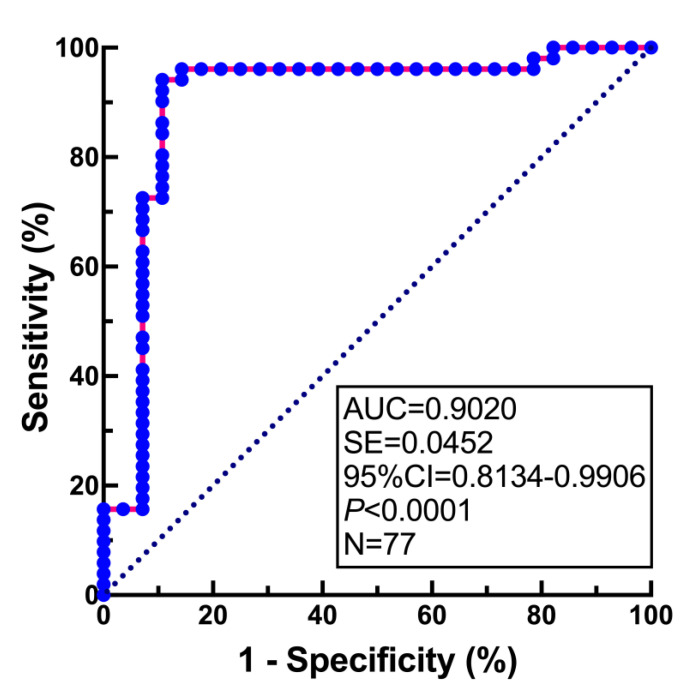
**ROC analysis of *FUT4* mRNA expression in the endometrium of women with endometriosis and control women.** The area under the curve (AUC), standard error (SE), 95% confidence interval (95% CI), *p*-value and the total number of analyzed samples (*n*) are given in an inset.

The discriminatory ability of *FUT4* mRNA expression in the total population analyzed was evaluated by ROC analysis. As can be seen in Figure 4, the AUC value was very high and was associated with high statistical significance (AUC = 0.90, 95% CI = 0.81–0.99, *p* < 0.0001). The diagnostic value of *FUT4* mRNA evaluation is summarized in Table 2. As can be seen, the relative expression of *FUT4* mRNA with a cutoff value of 0.002793 units (2^−ΔCt^) conferred 94.12% sensitivity and 89.29% specificity. The positive predictive value (PPV) and the negative predictive value (NPV) for the diagnosis of endometriosis were 93.9% and 89.3%, respectively. Youden’s statistics revealed a value of 0.8341.

## 4. Discussion

This study is the first to present data showing that *FUT4* mRNA levels are significantly upregulated in the eutopic endometrium of patients with endometriosis when compared to the endometrium of healthy control women. ROC analysis revealed that the expression of *FUT4* mRNA in samples of the eutopic endometrium could serve as a suitable marker for the minimally invasive diagnosis of endometriosis. These data also suggested that *FUT4* may play an important role in the etiopathogenesis of this disease.

The expression of *FUT4* in mammalian endometrium was reported to be specific for glandular and luminal epithelium [27,28,40,41], and as previously mentioned, it characterizes a population of endometrial glandular precursor/stem cells [27,28]. It has been suggested that *FUT4* expression in the endometrium may be upregulated by progesterone and thus related to the phase of the menstrual or estrus cycle [40,41,42,43]. Conversely, other studies in mice claimed that *FUT4* expression was suppressed by progesterone and stimulated by estrogen, indicating that the effects of both hormones are antagonistic [44]. In the present study, we did not discover any significant difference in *FUT4* mRNA expression in the endometrium when comparing control women to women with endometriosis in the mid-proliferating and mid-secretory phase. Similarly, no changes were observed in serum levels of SLeX, a product of *FUT4* activity, during the menstrual cycle in healthy women [45]. Inconsistent results for *FUT4* mRNA expression across the literature in the case of human material may be related to different methods of sample collection, sample origin, PCR method and other factors. Thus, the mechanisms that regulate *FUT4* expression during the menstrual cycle require further investigation.

Despite an increased number of SSEA-1^+^ cells, Hapangama et al. were unable to show increased expression of *FUT4* mRNA in the eutopic endometrium of women with endometriosis [28]. This may have been due to the limited number of patients since in the present study, we found that the expression of *FUT4* mRNA was markedly upregulated with very high statistical significance. A relationship between increased serum concentrations of SLeX and endometriosis was previously reported by Iwanari et al. [45,46]. However, increased serum concentrations of SLeX were also found in patients with ovarian [45,46] and breast cancer [47], meaning its diagnostic specificity appears to be limited. Our present results show that this problem may be circumvented by tissue-specific evaluation of *FUT4* mRNA. ROC analysis confirmed that the expression of *FUT4* mRNA in the eutopic endometrium is a specific marker of endometriosis. Evaluation of *FUT4* mRNA for the diagnosis of endometriosis fulfills the criteria of high accuracy, sensitivity and specificity with high positive and negative prediction values. Samples of endometrium may be easily obtained for mRNA evaluations by a minimally invasive aspiration biopsy during a visit to a gynecological office. Therefore, the evaluation of *FUT4* mRNA appears to be a suitable method for a minimally invasive diagnosis of endometriosis. This, however, will require further verification by an independent replicate study using different cohorts of patients.

Increased expression of *FUT4* mRNA in endometriosis was observed irrespective of the lesion type or location. Furthermore, there was a positive correlation between the levels of *FUT4* mRNA and the disease severity. This may support the role of FUT4 activity and *FUT4*-mediated glycosylation in the etiopathogenesis of endometriosis.

It is plausible that higher levels of *FUT4* mRNA in the eutopic endometriosis tissue simply reflect the persistence of increased numbers of SSEA-1^+^ precursor/stem cells. However, the levels may also be explained by increased expression of the *FUT4* gene in endometriosis-associated SSEA-1^+^ epithelial glandular cells [28]. The expression of fucosyltransferases and their activity are significantly increased in various tumors [48,49]; however, the mechanisms responsible for this phenomenon remain obscure. Possible stimulatory factors may include hypoxia [50], proinflammatory cytokines [51] or cell stress [52]. These factors are considered to play a part in the etiopathogenesis of endometriosis; therefore, it is plausible that they may contribute to increased expression of *FUT4* mRNA in the endometrium of women suffering from endometriosis. This hypothesis, however, needs to be further investigated.

In a variety of tumor cells, overexpression of *FUT4* is attributed to their increased proliferation rate, decreased susceptibility to apoptosis as well as increased adhesiveness, invasiveness and metastasis, and is generally related to a poor prognosis [48,49]. Interestingly, fucosylation may also be responsible for the modification of a variety of signaling molecules including TGF-β receptors that, in turn, may contribute to an increased rate of epithelial-to-mesenchymal transition and matrix metalloproteinases-related invasiveness [48]. It is not known whether similar effects may be observed in the course of endometriosis. However, it is intriguing to speculate that, in addition to being a marker of endometriosis, overexpression of *FUT4* may play an important role in the development and persistence of endometriotic lesions. In future research, a pathogenic role of *FUT4* in endometriosis awaits extensive investigation.

## 5. Conclusions

The results of the present study show that the expression of *FUT4* mRNA in the endometrium of patients with endometriosis is significantly increased when compared to healthy women. Therefore, *FUT4* mRNA from the endometrium may serve as a specific marker that can be used to diagnose endometriosis in a minimally invasive manner. Furthermore, it appears that *FUT4*-mediated fucosylation may play an important role in the development and persistence of endometriotic lesions and, therefore, may be considered a target for endometriosis therapies.

## Figures and Tables

**Table 1:** Demographic and clinical characteristics of patients with endometriosis and control subjects.

		Control Group			Endometriosis Group		
		All	Mid-Proliferative Phase	Mid-Secretory Phase	All	Mid-Proliferative Phase	Mid-Secretory Phase
Number of cases (N)		28	12 (42.9%)	16 (57.1%)	49	18 (36.7%)	31 (63.3%)
Age, years (mean ± SD)		35.2 ± 6.40	36.0 ± 5.02	34.5 ± 7.37	31.9 ± 5.53	29.4 ± 3.73	33.4 ± 5.93
BMI, kg/m^2^ (mean ± SD)		22.2 ± 4.79	22.8 ± 6.08	21.9 ± 3.96	21.7 ± 3.12	21.5 ± 2.44	21.8 ± 3.50
Parity		0.8 ± 0.96	1.0 ± 1.10	0.6 ± 0.84	0.35 ± 0.67 *	0.18 ± 0.39	0.45 ± 0.78
Infertility		0	0	0	18 (36.7%) **	9 (50.0%)	9 (29.0%)
rAFS	I (minimal)	-	-	-	8 (16.3%)	2 (11.1%)	6 (19.4%)
	II (mild)	-	-	-	6 (12.2%)	0 (0%)	6 (19.4%)
	III (moderate)	-	-	-	25 (51.0%)	11 (61.1%)	14 (45.1%)
	IV (severe)	-	-	-	10 (20.5%)	5 (27.8%)	5 (16.1%)
Lesion localization	Ovarian	-	-	-	37 (75.5%)	16 (88.9%)	21 (67.7%)
	Peritoneal	-	-	-	35 (71.4%)	13 (72.2%)	22 (71.0%)
	Both	-	-	-	23 (46.9%)	11 (61.1%)	12 (38.7%)

*p*-values were calculated by one-way ANOVA, Student’s *t*-test or the Chi-squared test when applicable. * Different from all control groups at *p* = 0.0200. ** Different from all control groups at *p* = 0.0058.

**Table 2:** Diagnostic value of *FUT4* mRNA expression in the eutopic endometrium to predict endometriosis.

Sensitivity (95%CI)	94.12% (84.08–98.40%)
Specificity (95%CI)	89.29% (72.80–96.29%)
Positive Predictive Value (PPV)	93.9%
Negative Predictive Value (NPV)	89.3%
Likelihood Ratio	8.784
Accuracy	92.2%
Youden’s Index	0.8341

The threshold for relative *FUT4* mRNA expression was established at 2^−ΔCt^ = 0.002793; 95% confidence interval (95%CI) values are given in parentheses.

## Data Availability

Specific data are available from the authors upon request.
